# Large orthotopic reservoir stone burden: Role of open surgery

**DOI:** 10.4103/0974-7796.68856

**Published:** 2010

**Authors:** Khaled Madbouly

**Affiliations:** Mansoura Urology and Nephrology Center, Mansoura, Egypt

**Keywords:** Bladder, late complications, orthotopic diversion, radical cystectomy, reservoir stones

## Abstract

**Purpose:**

To present our experience in open poucholithotomy as a primary management of large orthotopic reservoir stone burden and discuss different management options.

**Materials and Methods:**

Records of men underwent radical cystectomy and orthotopic urinary diversion were retrospectively reviewed as regards pouch stone formation. Patients with large reservoir stone burden managed by open poucholithotomy were further selected.

**Results:**

Large reservoir stone burden was encountered in 12 post radical cystectomy men. All underwent open poucholithotomy as a primary management of their reservoir stones. Median age at cystectomy was 46 (range: 32-55) years with a median total follow up period of 214.15 (range: 147-257) months and a median interval to stone detection of 99 (range: 63-132) months. The median stone burden was 5260 (range: 3179-20410) mm^2^. All patients were continent during the day while 5 showed nocturnal enuresis; 2 of them became continent after removal of the stones. Post poucholithotomy, all patients had sterile urine cultures except one who showed occasional colonization. None of the 12 patients showed stone recurrence after poucholithotomy. Two patients underwent revision of a dessuscepted nipple valve in association with stone removal.

**Conclusions:**

Open poucholithotomy for large reservoir stone burden is a feasible and safe option. It saves the reservoir mesentery and adjacent bowel. It allows complete removal of the stone(s) leaving no residual fragments. Furthermore, it permits correction of concomitant reservoir abnormalities.

## INTRODUCTION

Formation of calculi within orthotopic urinary reservoirs is a well-known late complication.[1–4] Urinary stasis due to impaired voiding, urinary infection with urea splitting organisms, encrustation on foreign bodies and exposed metallic staples, mucus as well as metabolic acidosis are all contributing factors.[1,5,6] Calculus formation has been reported in up to 16.3% of urethral hemi-Kock patients[2] and to a less extent in other reservoir patients.[3] Most of pouch stones could be managed endoscopically,[2–4] little is mentioned about the role of open surgery.

We report our experience in open surgical management of 12 men with large reservoir stone burden after different types of urinary diversion. To the best of our knowledge, this is the largest reported series with the longest follow-up.

## MATERIALS AND METHODS

Since 1986, we offered bladder cancer patients the option of orthotopic urinary diversion. Currently, we exceeded two thousand patients. The urethral hemi-Kock pouch has been our diversion of choice in patients undergoing cystectomy till the ileal w-shaped neobladder was introduced in 1992.

Patients were followed up every 3 months during the first year and 6-monthly thereafter. The follow up included a history, physical examination, urine culture and blood chemistry studies. A base-line intravenous urography and an ascending pouchogram study were performed at first follow up followed by a yearly renal ultrasonography and/or intravenous urography. Computerized tomography and bone scans were used as clinically indicated. Continence was assessed by interviewing the patients at each follow up visit. They were considered to be continent if they were completely dry during the day and night with no need for protection by pads, condom catheter or medication.

All male patients’ records were retrospectively reviewed as regards late complications specially pouch stone formation. Male patients with large reservoir stone burden who were managed with open poucholithotomy represent the subject of this review. Stone length and width were measured in the maximum diameters and stone surface area was estimated.[7] In cases of multiple stones, the sum of the surface area of individual stones was determined.

One day preoperatively, patients were maintained on oral neomycin, metronidazole and a low residual or fluid diet. Intravenous fluids were given the night before surgery to maintain hydration. A third-generation cephalosporines and metronidazole were started half an hour before surgery and continued for 3-5 days postoperatively. Surgery comprised a transperitoneal approach through the previous cystectomy scar. Extra care was taken in dissection and mobilization of intestinal loops and pouch mesentery. An anterior pouchotomy of sufficient length was performed with dissection of the stone from the pouch wall or valve staples. Refixation of a dessuscepted valve was carried out in 2 patients. The pouchotomy was closed with continuous polyglycolic acid sutures leaving a draining urethral catheter for 2 weeks. In patients with valve revision, 2 ureteral stents passing through the valve into the pouch were used for 12-14 days and the urethral catheter was left for 3 weeks.

## RESULTS

By the end of 2007, radical cystectomy and urinary diversion were performed in 3933 patients. Number of patients and types of urinary diversion utilized are given in Table 1.

**Table 1:** Types of urinary diversion

Type of diversion	Number	(%)
Ileal loop conduit	1308	(33.3)
Orthotopic reservoirs		
Ileal w-neobladder	1271	(32.3)
Urethral hemi-Kock	353	(9)
Others		
Continent cutaneous diversion	174	(4.4)
Rectal diversion	827	(21)
Total	3933	(100)

Pouch stones were present in 15.6% (55 out of 353) patients with urethral hemi-Kock pouch. The stones recurred once in 10 patients, twice in 2 and for the third time in one patient. Twenty nine (2.3%) out of 1271 patients with w-neobladder developed reservoir stones. All stones but 12 were managed endoscopically.

Open poucholithotomy of large reservoir stone burden was performed in 12 post radical cystectomy men with a median age at cystectomy of 46 (range: 32-55) years, 10 with urethral hemi-Kock reservoirs and 2 with w-neobladder. Two patients with urethral hemi-Kock pouch underwent simultaneous revision of a dessuscepted valve.

Patients were followed for a median of 214.15 (range: 147 – 257) months.

**Figure 1:**
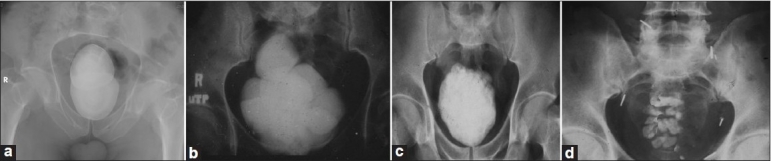
Large orthotopic reservoir stones. (a-c) Hemi-Kock pouch reservoirs. Staple lines could be seen on either side of the stone(s); (d) Ileal w-neobladder reservoir

The interval to stone detection varied from 61 to 132 months with a median of 99 months. The median stone burden was 5260 (range: 3179-20410) mm^2^ [Figure 1].

All patients were continent during the day while 5 showed nocturnal enuresis; 2 of them became continent post poucholithotomy. The remaining 3 patients showed less frequent episodes of nocturnal enuresis and a better response to antienuretic medications. Urine cultures were persistently sterile in 4 patients, persistently colonized in 3 (*Morganella morganii and E-coli*) and occasionally colonized in 5 (*Morganella morganii, E-coli* and *Serratia marscecenes*) [Table 2]. Post poucholithotomy, all patients had sterile urine cultures except one who showed occasional colonization (*Morganella morganii*). Radiologically, the upper urinary tract was maintained as preoperatively in 20 renal units, improved in 3 and deteriorated in one unit (patient with w-neobladder) with no effect on the serum creatinine. None of the 12 patients showed stone recurrence after poucholithotomy.

**Table 2:** Details of patients pre- and post-poucholithotomy

Patient	Age at cystectomy (years)	IVU		Time to stone detection (months)	Continence pre poucholithotomy		Urine culture pre poucholithotomy	Continence post poucholithotomy		Urine culture post poucholithotomy	Follow up post poucholithotomy (months)
		R RU	L RU		Day	Night		Day	Night		
1	32	Improved	Stable	66	+	+	Sterile	+	+	Sterile	257
2	55	Stable	Stable	63	+	+	Colonized	+	+	Sterile	229
3	45	Improved	Stable	132	+	+	Colonized	+	+	Sterile	254
4	54	Stable	Stable	120	+	–	Sterile	+	+	Sterile	165
5	45	Stable	Improved	108	+	+	Occasionally colonized	+	+	Sterile	230
6	47	Stable	Stable	108	+	+	Occasionally colonized	+	+	Sterile	155
7	48	Stable	Stable	99	+	–	Occasionally colonized	+	–	Sterile	206
8	45	Stable	Stable	90	+	–	Sterile	+	+	Sterile	227
9	42	Stable	Stable	88	+	+	Occasionally colonized	+	+	Occasionally colonized	222
10	48	Stable	Stable	80	+	–	Occasionally colonized	+	–	Sterile	203
11*	45	Stable	Deteriorated	106	+	+	Sterile	+	+	Sterile	168
12*	51	Stable	Stable	98	+	–	Colonized	+	–	Sterile	147

* Patients with ileal w-neobladder reservoir; IVU - Intravenous urography; RRU - Right renal unit; LRU - Left renal unit

## DISCUSSION

Orthotopic urinary diversion is believed to offer the best alternative with respect to bladder substitution when cystectomy is required.[8,9] However, it is not free of early and late complications. A pouch-related late complication rate between 11.6% and 23.5% has been reported in different series.[2–4,8,10]

Reservoir calculus formation is a well-known late complication. Factors contributing to stone formation in patients with intestinal urinary diversion are complex and incompletely understood. They include urinary stasis caused by impaired voiding with subsequent residual urine, urinary infection specially with urea splitting organisms and encrustation and stone formation on foreign bodies as exposed staples used for fixation of intussuscepted nipple valves.[5] Mucus produced by the intestine may also play a role. Small crystals can adhere to mucus, which could augment crystal retention and aggregation. It is also hypothesized that mucus may serve as a template for bacterial biofilm formation.[11]

Diversion patients have been known to have mild metabolic acidosis due to bicarbonate loss and reabsorption of urinary solutes including ammonium chloride and hydrogen ions. Metabolic acidosis promotes bony demeneralisation via a buffering process which liberates calcium and increases calcium excretion. Acidosis also inhibits calcium absorption in the nephron which also accentuates calcium excretion. Furthermore, excess ammonium absorption enhances intestinal sulfate uptake and renal sulfate excretion in this patient group which increases the filtered load of sulfate to the nephron and inhibits calcium reabsorption. Acidosis also induces hypocitraturia which is a known risk factor for calcium stone formation.[1,6] It has been reported that the continent urinary reservoirs cause a long-term increase in urinary excretion of calcium, phosphate and magnesium which together with hpocitraturia can contribute to stone formation.[12]

Because of the increased risk of urolithiasis in patients with orthotopic urinary diversion, a life-long follow up is recommended. However, after being free of significant complications for a long period, patients become less compliant for follow up protocols with increased length of periods between follow up visits which permits the development of large reservoir stones. This was exactly the case in our patients. The median interval to stone detection in our patients was 99 months. Furthermore, 6 of the 12 patients are working in Gulf countries.

Most of pouch stones either pass spontaneously or are retrieved endoscopically.[2,8] Extracorporeal shock wave lithotripsy (ESWL) was also reported as a successful treatment of pouch stones in the prone position.[13]

A percutaneous approach was considered for patients with continent pouches harboring large stones. However, CT should be used to guide the access in these patients to avoid injuring the mesenteric blood supply of the reservoir or adjacent bowel. The pouch should be distended with sterile saline and the access puncture should be performed at the point where the pouch is closest to the abdominal wall. A large diameter Foley catheter should be placed through the access tract into the reservoir after stone removal to ensure good drainage and left indwelling for a week. Upon removal of this catheter, a 16 F Foley catheter should be placed through the stoma into the reservoir to facilitate closure of the tract and prevent urinary extravasation.[14]

More recently, use of laparoscopic trocars was described with the introduction of a laparoscopic entrapment sac.[15] The stones are placed in the sac and a small incision is made for intact stone delivery or lithotripsy is carried out within the sac. Requirement for multiple ports, CT guidance, need for a retrieval incision, necessity for closure of the pouch and fascia as well as high experience in such manoeuvers are obviously limiting factors.

The situation in orthotopic urinary reservoirs is considerably different and more complex. They are located deep in the pelvis and not fixed to the abdominal wall. The omentum, bowel and the mesenteric blood supply of the reservoir separate it from the abdominal wall, making percutaneous approach not preferable or even dangerous. Alternatively, the large stone burden precludes the option of ESWL. Although endourological management has been the mainstay of treatment for calculi within a urinary reservoir,[2,3,15,16] in such large reservoir stone burden it will be associated with prolonged manipulations through the urethra which may endanger the sphincter mechanism and subsequently the state of continence or may be complicated by urethro-ileal contracture or urethral stricture. Furthermore, open poucholithotomy allows correcting associated abnormalities like revision of a nipple valve, reduction of a large pouch or reimplantaion of a ureter in case of ureteroileal stenosis.

We believe that poucholithotomy remains an excellent option for managing patients with large reservoir stone burden. It keeps the integrity of the pouch and the sphincteric mechanism as well as it preserves the continence status.

## CONCLUSIONS

Patients with urinary diversion are at increased risk of urolithiasis. Open poucholithotomy is an excellent option for primary management of large reservoir stone burden. It saves the reservoir mesentery and adjacent bowel. It allows complete removal of the stone(s) leaving no residual fragments with subsequent decrease of stone recurrence. It preserves the integrity of the pouch and sphincteric mechanism as well as the continence status. Furthermore, it permits correction of concomitant reservoir abnormalities.
